# Inhibitory Role of Berberine, an Isoquinoline Alkaloid, on NLRP3 Inflammasome Activation for the Treatment of Inflammatory Diseases

**DOI:** 10.3390/molecules26206238

**Published:** 2021-10-15

**Authors:** Paromita Sarbadhikary, Blassan P. George, Heidi Abrahamse

**Affiliations:** Laser Research Centre, Faculty of Health Sciences, University of Johannesburg, Doornfontein 2028, South Africa; paromitas@uj.ac.za (P.S.); habrahamse@uj.ac.za (H.A.)

**Keywords:** apoptosis, berberine, diseases, inflammation, NLRP3 inflammasome, phytochemical, signaling pathways, therapeutics

## Abstract

The pyrin domain-containing multiprotein complex NLRP3 inflammasome, consisting of the NLRP3 protein, ASC adaptor, and procaspase-1, plays a vital role in the pathophysiology of several inflammatory disorders, including neurological and metabolic disorders, chronic inflammatory diseases, and cancer. Several phytochemicals act as promising anti-inflammatory agents and are usually regarded to have potential applications as complementary or alternative therapeutic agents against chronic inflammatory disorders. Various in vitro and in vivo studies have reported the anti-inflammatory role of berberine (BRB), an organic heteropentacyclic phytochemical and natural isoquinoline, in inhibiting NLRP3 inflammasome-dependent inflammation against many disorders. This review summarizes the mechanism and regulation of NLRP3 inflammasome activation and its involvement in inflammatory diseases, and discusses the current scientific evidence on the repressive role of BRB on NLRP3 inflammasome pathways along with the possible mechanism(s) and their potential in counteracting various inflammatory diseases.

## 1. Introduction

Inflammation is an evolutionarily conserved defense response of the immune system to protect the host against harmful stimuli such as bacteria, viruses, toxins, contaminants, and tissue injury. However, the past two decades have witnessed widespread research on inflammation not because of its protective role but due to the involvement in a variety of diseases that make up highest percentages of morbidity and mortality worldwide. Several environmental, social, and lifestyle factors lead to long-term systemic chronic inflammation, which alters normal cellular physiology in all tissues and organs. Severe chronic inflammation, in turn, increases the risk of developing a myriad of mental, physical, and metabolic disorders such as cardiovascular disease, cancer, diabetes mellitus, hypertension, nonalcoholic fatty liver disease, chronic kidney disease, and autoimmune and neurodegenerative disorders [1,2,3].

Inflammasomes that are included in the nucleotide binding domain (NOD)-like receptor (NLR) family belongs to the second category of pattern recognition receptors (PPRs) that responds to intracellular cytosolic disturbances. The term inflammasome was first coined by Tschopp et al., in 2002, to define a multiprotein complex that is activated in response to several pathogen-associated molecular patterns (PAMPs) and damage-associated molecular patterns (DAMPs) signals. Further, its stimulation results in the processing and activation of pro-caspase-1, which promotes the production and secretion of pro-inflammatory cytokines such as interleukin-1β (IL-1β) and IL-18 and induces cell death via pyroptosis [4,5]. Conversely, the secretion of excess pro-inflammatory cytokines is associated with several human diseases ranging from cancer to autoinflammatory, cardiovascular, neurological, and metabolic disorders. Further, the uncovering of the fact of the involvement of inflammasome function in common human pathologies has encouraged the basic and translational research on inflammasomes as a potential therapeutic target for several disorders [6,7,8,9]. Up until now, none of the drugs have received approval for its efficacy against the uncontrolled activation of the NLRP3 inflammasome [10].

Several plant-based phytocompounds have shown promising anti-inflammatory properties that make them potential candidates in complementary therapy against several chronic human pathologies caused by severe inflammation [10,11]. One such phytochemical is berberine (BRB). BRB is an isoquinoline alkaloid usually isolated from a medicinal herb *Rhizoma coptidis*, which suppresses intracellular reactive oxygen species (ROS) generation and pro-inflammatory responses by inhibiting several inflammation-associated signaling pathways [10,11,12]. Recently, BRB has been shown to exert its beneficial anti-inflammatory effect by modulating NLRP3 inflammasome expression and activity [10,12]. Therefore, this review mainly aimed to provide recent evidence of the inhibitory role of BRB on the NLRP3 inflammasome pathway alongside reported molecular mechanism(s). The review also discusses the activation and regulation mechanisms of the NLRP3 inflammasome with its implications in the pathogenesis of several chronic inflammatory diseases.

## 2. NLRP3 Inflammasome Activation and Its Role in Inflammatory Disorders

Inflammasomes are intracellular cytoplasmic multi-protein complexes that are expressed primarily in monocytes and macrophages and, to a certain extent, in dendritic cells and neutrophils, as well as in some nonimmune cells, such as keratinocytes, smooth muscle cells, and endothelial cells [13]. Inflammasomes constitute a fundamental role in the host’s innate immune response to infections and internal cellular injuries and are involved in PAMPs and DAMPs sensing and the regulation of their downstream signaling. The inflammasome acts via pro-caspase-1 activation, which, in turn, promotes the secretion of mature pro-inflammatory cytokines IL-1β and IL-18, as well as promoting pyroptosis, an inflammatory form of cell death [4,14,15]. The reported multimeric inflammasomes are classified based on their sensors, which include the members of both the NLR family comprising NLR pyrin domain (PYD)-containing NLRP1, NLRP3, NLRC4, NLR family CARD (caspase activation and recruitment domain), and NAIP (NLR family apoptosis inhibitory protein containing 4), and the non-NLR family such as AIM2 (absent in melanoma 2), as well as Pyrin inflammasomes [16].

### 2.1. NLRP3 Inflammasome

**Figure 1:**
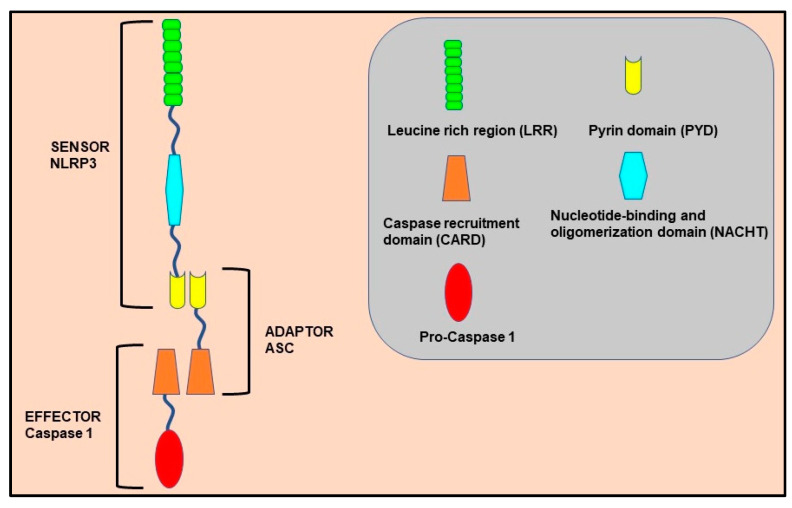
Domain structure and assembly of the NLRP3 inflammasome multicomplex. NLRP3 and ASC interact with their PYD domains to activate the complex, whereby the pro-caspase 1 protein is further recruited to the complex via their CARD domain. This interaction cleaves and forms the catalytically active caspase-1. NLRP3: nucleotide-binding domain (NOD)-like receptor protein; ASC: apoptosis-associated speck-like protein containing a CARD.

NLRP3 is one of the most well-characterized and extensively studied inflammasome complexes, which was first reported to be linked with hereditary autoinflammatory disease and cryopyrin-associated periodic syndromes. As shown in Figure 1, this multi-protein complex comprises NLRP3 as a sensor, ASC (apoptosis-associated speck-like protein containing a CARD, also known as PYCARD) as an adaptor, and caspase 1 as an effector. The NLRP3 gene encodes for the tripartite motif protein, NLRP3, containing an N-terminus PYD domain, a central NOD or NACHT domain, and a leucine-rich repeat (LRR) as a C-terminus domain [15]. The ATPase activity of the central NACHT domain is crucial for NLRP3 oligomerization and pro-caspase-1 activation, while the LRR domain induces autoinhibition via its interaction with the central NACHT domain. The adaptor ASC consists of two protein-interacting domains: an N-terminus PYD, which interacts with the PYD domain of the NLRP3 protein; and a C-terminus CARD, which binds and activates pro-caspase 1. Besides its N-terminus CARD, pro-caspase 1 also consists of large (p20) and small (p10) catalytic subunits at its central and C-terminus domains. The NLRP3 inflammasome responds to a diverse range of pathogen-derived infectious signals PAMPs, as well as endogenous danger signals DAMPs [4,15,17,18].

### 2.2. Activation of NLRP3 Inflammasome via Host-Derived Activating Signals

**Figure 2:**
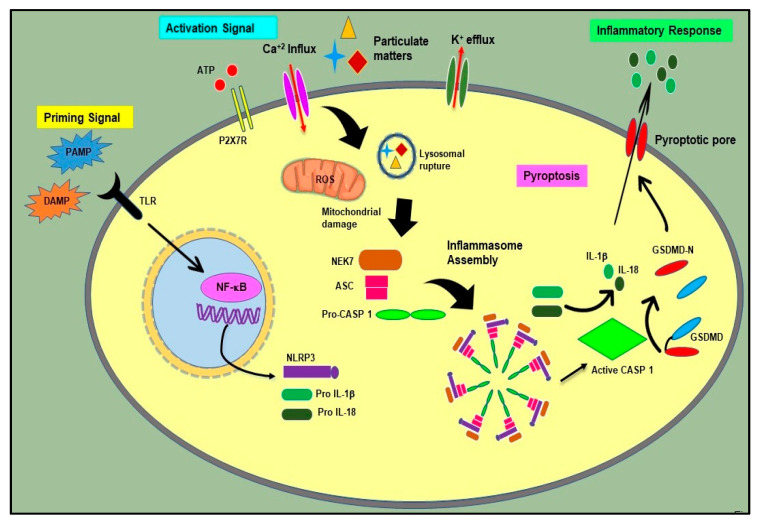
Schematic representation of NLRP3 inflammasome priming and activation. As part of the priming signal 1, pathogen-associated molecular patterns (PAMPs) and danger-associated molecular patterns (DAMPs) bind and activate Toll-like receptors (TLRs), resulting in NF-κB-mediated upregulation of NLRP3 inflammasome components and precursors of inflammatory cytokines. Meanwhile, particulate matter, crystals, ATP, toxins, lysosomal disruption, K^+^ efflux, mitochondrial ROS generation, Ca^2^^+^ influx, etc. act as triggers for activation signal 2 and induce the assembly of NLRP3, ASC, and pro-caspase 1 into an active NLRP3 multicomplex inflammasome. The formation of an activated inflammasome causes the activation of caspase-1, which, in turn, cleaves and activates pro IL-1β and pro IL-18 into its mature IL-1β and IL-18 forms, which are secreted from the cells, inducing an inflammatory response. Further, the active caspase-1 catalytically cleaves and activates the pore-forming protein Gasdermin D (GSDMD), resulting in pore formation in the cell membrane and inducing pyroptosis.

Host-derived endogenous signals (DAMPs) such as ROS generation, cell volume changes, K^+^ ion efflux, Ca^2^^+^ signaling, mitochondrial dysfunction, and lysosomal disruption trigger the activation and regulation of NLRP3 inflammasomes. Tight regulation of the inflammasome signaling pathway is necessary to prevent the unrestrained stimulation of associated inflammatory responses to restrict the unwarranted damage in the host body [4]. Activation of the inflammasome occurs in two steps—the first signal, is priming, followed by the second signal activation, as represented in Figure 2. In the priming step, PAMPs, DAMPs, and other cytokines such as IL-1β and tumor necrosis factor (TNF) stimulate and induce the NF-κB-mediated upregulation of NLRP3 inflammasome components, such as NLRP3 proteins, inactive pro-caspase 1, pro-caspase-4/5 or pro-caspase 8, pro-IL-1β, and pro-IL-18 [17,18,19]. Subsequently, various DAMPs such as ATP, toxins, particulate molecules, and the influx or efflux of ions act as the second activation signal, which triggers the activation and formation of the NLRP3 inflammasome by activating multiple upstream signaling pathways. Upon stimulation, homotypic interactions between NACHT domains result in the oligomerization of NLRP3. Oligomerized NLRP3 then recruits ASC through homotypic interactions between PYD domains of NLRP3 and ASC, which nucleates helical ASC filament formation. Multiple ASC filaments unite to form single macromolecular distinct foci, known as ASC speck. The formation of ASC speck causes the recruitment of pro-caspase 1 to the complex through CARD interactions [15,17,19], following which the ATPase activity of the NAHCT domain of the NLRP3 protein induces autocatalytic cleavage in Pro-caspase 1 to generate proteolytically active subunits p10 and p20, which remain bound to the assembly and form an active multicomplex inflammasome. The activated inflammasome subsequently proteolytically cleaves pro-IL-1β and, pro-IL-18 to their active form, along with the activation of cleaved Gasdermin D (GSDMD). Activated GSDMD is a pore-forming protein, which induces pyroptosis [15,19,20].

Both post-transcriptional and post-translational regulatory mechanisms are involved in the regulation of NLRP3 inflammasome activation [18,19]. Several microRNAs such as MiR-7, MiR-22, MiR-30e, and MiR-223 target the 3′-untranslated region (UTR) of NLRP3 mRNA, thus reducing the expression of the NLRP3 protein and suppressing IL-1β production [21], while long noncoding RNAs such as ANRIL, MALAT1, Neat 1, and Gm15441 have shown to be involved in either promoting or inhibiting the inflammasome signaling. The NLRP3 protein undergoes post-translational modifications at different sites and domains via phosphorylation, dephosphorylation, ubiquitination, de-ubiquitination, sumoylation, and S-nitrosylation, which occurs in response to either activating or inhibitory effectors [4,15,22]. Several molecules such as BCAP, IRGM, DDX3X and BHB (targeting K^+^ efflux), TMEM176B (targeting intracellular Ca^2^^+^), macrophage CGI-58, IL-10, and nitric oxide (NO) (targeting mitochondrial function) act as negative mediators and interfere with the NLRP3 inflammasome assembly and activation [4].

### 2.3. NLRP3 Inflammasome Associated Human Disease

Dysregulated and unwarranted activation of the NLRP3 inflammasome is involved with the initiation and development of several human disorders such as cardiovascular diseases, metabolic pathologies, neurological disorders, and inflammatory-related disease and complications, as listed in Table 1 [8,23,24,25]. Other than bacterial infections, hypoxia, ROS, extracellular ATP, and several disease-related proteins cause inappropriate activation of the NLRP3 inflammasome, leading to several disorders represented in Table 1.

### 2.4. Role of the NLRP3 Inflammasome in Cancer

The association of an inappropriately activated NLRP3 inflammasome with various auto-immune and inflammatory diseases, and neurodegenerative and metabolic disorders are well studied. However, studies have shown that the stimulation of inflammasomes have an opposing role in cancer pathogenesis, i.e., either being a tumor-suppressor by inducing tumor cell death, or a tumor-promoter by enhancing the production of pro-inflammatory cytokines by tumor cells and/or tumor-infiltrating stromal cells, within the tumor microenvironment [26]. The pro-tumorigenic role of the NLRP3 inflammasome has been reported in several different cancers such as breast, melanoma, head and neck squamous cell carcinomas, colorectal, lung, and hepatocellular carcinomas. Pro-tumorigenic effects of the NLRP3 inflammasome are mainly either due to the activation of exogenous stimuli-induced unusual inflammasomes and the simultaneous over-secretion of IL-1β and IL-18 and/or due to their constitutive expression and activation even without the presence of any exogenous stimuli. Mainly IL-1β and, to a lesser extent, IL-18 have been shown to be involved in tumor proliferation, survival, metastasis, angiogenesis, and immunosuppression, thus promoting the development and progression of cancers. The increase in the expression of adhesion molecules, such as intercellular adhesion molecule-1 (ICAM-1) in mesenchymal cells and vascular cell adhesion molecule-1 (VCAM-1) in endothelial cells, mainly occurs due to high IL-1β levels. This, in turn, promotes various inflammatory cells such as myeloid-derived suppressor cells (MDSCs) and tumor-associated macrophages (TAMs) to infiltrate at the tumor site. This establishes an inflammatory microenvironment, which promotes cancer progression and metastasis. The tumor-infiltrating immune cells, such as neutrophils, dendritic cells, and TAMs, further promote the secretion of IL-1β in the tumor microenvironment [27]. IL-1β secretion also contributes to angiogenesis by promoting the release of cytokines such as vascular endothelial growth factor (VEGF), macrophage-inflammatory protein-2 (CXCL2), and hepatocyte growth factor (HGF). Further, IL-1β regulates hypoxia in the tumor mass by suppressing miR-101 expression through the COX2-HIF1α pathway. IL-1β has also shown to promote proliferation in cancer cells by activating several oncogenic signaling pathways [8,28,29,30]. In addition, extracellular ATP released from damaged tumor cells upon anti-cancer treatment also induces NLRP3 inflammasome activation [27]. Studies have suggested the role of IL-18 in the polarization of T cells toward Th1 or Th2 cells by inducing IL-17 expression in Th17 cells in certain types of cancer, while the role of IL-18 in tumor progression has not been fully elucidated yet [27]. Further, IL-18 has been shown to promote tumorigenesis in later stages due to its involvement in downregulating the soluble IL-22 receptor and IL-22-binding protein (IL-22BP), thus raising the IL-22/IL-22BP ratio [26]. However, further studies are underway to reveal the better depiction of the pathophysiological role of NLRP3-IL-1β/IL-18 in tumorigenesis.

Besides the direct role of aberrant stimulation of the NLRP3 inflammasome, few studies have recently also suggested that the dysregulated interaction between the inflammasome and autophagy can be linked with the development and progression of cancer. Autophagy is considered to inhibit cancer progression by mitochondrial ROS clearance, and the removal of misfolded proteins and dysfunctional organelles, all of which act as stimuli for overactivation of the inflammasome. Further, autophagy is also involved in the degradation of inflammasome components and sequestration of pro-IL-1β. Therefore, excessive mitochondrial oxidative stress caused by defective autophagy results in aberrant activation of the inflammasome, and thus promotes tumorigenesis, cancer stemness, metastasis, and therapeutic resistance [30,31]. Additionally, NLRP3 genetic disorders also lead to inherited autoinflammatory disorders. Autoinflammatory disorders are inherited diseases that are characterized by unprovoked occurrences of systemic and organ-specific inflammation attributed by the defects in the innate immune system. Gain-of-function mutations in the NLRP3 gene have been directly linked to the related manifestation of autoinflammatory disorders, which leads to the amplified release of IL-1β. The first autoinflammatory disorders reported were a group of illnesses collectively known as cryopyrin-associated periodic syndromes (CAPSs). CAPSs include chronic infantile neurological cutaneous and articular syndrome (CINCA), familial cold autoinflammatory syndrome (FCAS), and Muckle–Wells syndrome (MWS) [24,32]. Recent and upgraded findings in the area of NLRP3 inflammasome biology, and its role and regulation in several autoimmune and inflammatory disorders and diseases have highlighted its potential as a novel therapeutic target. Thus, so far, various inhibitors of the NLRP3 inflammasome pathway have been identified and substantiated in numerous in vitro and in vivo inflammatory disease models. Some of these inhibitors, represented in Table 2, directly target the NLRP3 inflammasome protein complex, while others indirectly target other constituents and products of the inflammasome pathway [33,34,35,36,37].

## 3. Berberine

The use of berberine (BRB) as traditional Ayurvedic and Chinese medicine dates to more than 3000 years ago. Crude extracts and decoctions of bark, roots, and stems of numerous plants (Table 3) rich in berberine have been extensively used as treatment remedies against several ailments, including infectious diseases, inflammation, diabetes, constipation, diarrhea, cancer, and other pathologies [38].

### 3.1. Chemistry and Pharmacokinetics

BRB, chemically known as 5,6-dihydro-9,10-dimethoxybenzo[g]-1,3-benzodioxolo [5,6-a] quinolizinium, is a nonbasic and quaternary ammonium benzylisoquinoline alkaloid [39]. The active compound of BRB, protoberberine, exists as a mixture of three tautomeric forms, which are in equilibrium in nature. Protoberberine interconverts between its salt and base forms, their water-soluble salt forms are stable in acidic and neutral media, while the base form solubilizes in organic solvents. The most commonly available salt form of BRB is yellow-colored Berberine hydrochloride with a chemical formula of C20H18ClNO4, with a melting point of 145 °C. Further, the BRB is highly sensitive to heat and light, which influence its extraction methods. Extraction methods of BRB include both classical and modern techniques: classical techniques involve maceration, percolation, Soxhlet, and cold or hot continuous extraction, while the modern techniques make use of ultrasound, microwaves, ultrahigh pressure, supercritical fluid, and pressurized liquid-based extractions [38,39]. BRB has an apparent permeability coefficient of ~10^−7^ cm/s across the intestinal tissue. The poor bioavailability and low absorption of BRB are mainly due to P-glycoprotein expression in intestinal cells and CYP 450-dependent first-pass metabolism. Several metabolites of BRB have been reported in humans after its oral administrations such as demethyleneberberine2-*O*-sulfate, thalifendine, thalifendine-10-*O*-beta-d-glucuronide, jatrorrhizine-3-*O*-sulfate, jatrorrhizine-3-*O*-beta-d-glucuronide, berberrubine-9-*O*-beta-d-glucuronide, 3,10-demethylpalmatine-10-*O*-sulfate, columbamin-2-*O*-beta-d-glucuronide, and demethyleneberberine-2,3-di-*O*-beta-d-glucuronide [40,41].

### 3.2. Pharmacological Activities of BRB

BRB being one of the ancient phytomedicines exhibits a wide array pharmacological effects that make it a potential drug against a broad spectrum of human diseases. Several reviews have extensively discussed the action of BRB, its molecular targets, affected genes, signaling pathways, and cellular mechanism with its implications in various treatments [39,42,43,44,45,46,47]. Therefore, we provide here an overview of the potential pharmacological activities of BRB.

- BRB has shown to exert diverse effects in intracellular oxidative stress. Many studies have demonstrated its antioxidant activity whereby it has been shown to induce the scavenging of ROS/RNS, chelation of metal ions, increase in antioxidant effects of endogenous substances, superoxide dismutase activity, and decrease in lipid peroxidation [39]. On the contrary, BRB has also been shown to increase ROS production, which subsequently activated several apoptotic signaling pathways such as JNK, ERK1/2, and MAPK, as well as Akt and Ca^2^^+^-dependent pathways [42].
- The nucleic acid affinity of BRB has been proposed to be partially responsible for its anticancer efficacy. BRB binds strongly with DNA with more affinity for polyadenylic acid [poly(A)] than other polynucleotides [48]. The binding of BRB with nuclear DNA has been shown to induce dsDNA breaks, leading to activation of the p53-dependent pathway, inducing cell cycle arrest and apoptosis [49]. Similarly, BRB binding to mRNA inhibits the process of translation and translocation [42].
- BRB also inhibits several carcinogenesis-related enzymes by directly inhibiting cytosolic arylamine *N*-acetyltransferase, telomerase, and topoisomerase activity, thus acting as a potent topoisomerase poison. BRB also suppresses the translational and transcriptional expression of cytosolic arylamine *N*-acetyltransferase and cyclooxygenase-2 [48,50].
- BRB induces both p53-dependent G1 arrest and p53-independent G2/M cell cycle arrest. BRB suppresses the MDM2 inhibitor protein at the post-transcriptional level, thus upregulating p53 expression, leading to G1 arrest and apoptosis. G1 arrest has also been associated with the BRB-induced overexpression of CIP1/p21 and Kip1/p27 proteins and downregulation of cyclin-dependent kinases (cdk2, cdk4, and cdk6), while BRB-induced G2/M arrest is associated with the downregulation of cyclin B1 and upregulation of Wee1, leading to apoptotic cell death [43]. Thus, BRB has the potential to induce tumor cell death irrespective of their p53 status [50].
- BRB has been shown to induce apoptotic cell death by affecting several apoptotic signaling pathways, including p53-dependent and -independent, caspase-dependent mitochondrial, Fas receptor-dependent, and caspase-independent pathways [42,50]. BRB has also been shown to induce apoptosis by targeting the redox/ROS and JNK/p38 signaling pathways [51], as well as two pro-apoptotic proteins: ATF3 mediated in a p53-dependent manner and NAG-1 involving several pathways, PKC, ERK, and GSK-3β [52].
- The anti-inflammatory activity of BRB is mediated by the inhibition of the pro-inflammatory NF-kB pathway, which is attributed to the inhibition of IκB kinase (IKK) activation, causing the stabilization of IκB alpha, leading to the dephosphorylation and nuclear translocation of p65, and finally inhibiting the reporter activity of NF-κB. The inhibition, in turn, resulted in the suppression of NF-κB-regulated gene products involved in anti-apoptosis (Bcl-XL, Survivin, IAP1, IAP2, and cFLIP), proliferation (cyclin D1), inflammation (COX-2), and invasion (MMP-9) [53].
- The anti-invasion or anti-metastatic potential of BRB in cancer is mainly ascribed to the downregulation of nuclear transcription factors: c-fos, c-jun, and NF-κB; inhibition of TNF-α-induced MMP-9 expression, suppression of MMP-1, -2, and -9 and u-PA, through MAPK and NF-αB signaling pathways; and inhibition of the RhoA signaling pathway [48,50].

### 3.3. Inhibitory Action of Berberine on Inflammasome Pathway and Associated Diseases

**Figure 3:**
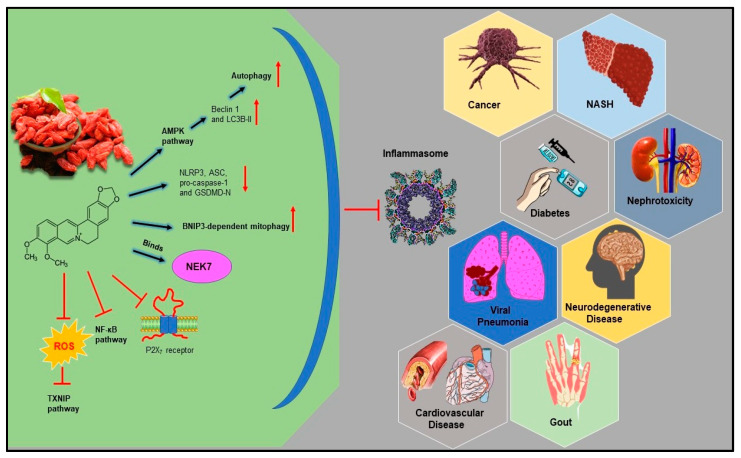
Reported molecular mechanisms of BRB (berberine) in NLRP3 inflammasome suppression in different inflammatory-related diseases: cancer, Nonalcoholic Steatohepatitis (NASH), nephrotoxicity, gout, viral pneumonia, Type II diabetes mellitus, and neurodegenerative and cardiovascular disease.

As discussed, BRB imparts its beneficial effect in several inflammatory and metabolic diseases via various mechanisms. However, even after its well-established anti-inflammatory potential, the effect of BRB on NLRP3 inflammasome stimulation and downstream pathways has not yet been understood. Therefore, currently, several research groups are actively involved in exploring the potential role and underlying mechanism(s) of BRB in triggering the NLRP3 inflammasome and induction of pyroptosis. Figure 3 summarizes the molecular mechanisms of BRB in NLRP3 inflammasome suppression reported for different inflammatory diseases.

#### 3.3.1. Cancer

To investigate the role of BRB on cancer cells, Yao et al. showed that the treatment of MDA-MB-231, a triple-negative breast cancer cell line, with BRB results in a significant decrease in the release of pro-inflammatory cytokines such as IL-1α, IL-1β, IL-6, and TNF-α, which are usually involved in tumor proliferation, progression, and metastasis [54]. BRB also downregulated P2 × 7 receptor expression, which acts as an activating trigger for the NLRP3 inflammasome. Further, BRB treatment resulted in the downregulation of mRNA and protein expression of NLRP3 inflammasome components. This leads to the inhibition of NLRP3 inflammasome activation and eventually leads to the decreased activity of the catalytic domain (p20) of caspase-1 and the suppression of IL-1β and IL-18 levels [54].

#### 3.3.2. Liver Disease

To evaluate the protective role of BRB in Nonalcoholic Steatohepatitis (NASH), a study demonstrated its inhibitory role on NLRP3 inflammasome activation and the subsequent pyroptosis process. Mechanistically, BRB was shown to significantly reduce the expression of NLRP3, pro-caspase 1, and GSDMD protein, along with decreasing the caspase-1 activity via the ROS-TXNIP pathway by inhibiting intracellular ROS in methionine-choline-deficient/lipopolysaccharide or palmitic acid-stimulated hepatocytes [55]. Another study reported by Vivoli et al. further proved the anti-inflammatory potency of BRB in two different in vivo models of liver injury caused by NASH. An underlying molecular mechanism study in LPS-stimulated macrophages showed the downregulated expression of NLRP3 inflammasome components mediated via interference with the purinergic receptor P2X_7_ [56].

#### 3.3.3. Macrophages and Macrophages-Mediated Diseases

The outcome of BRB treatment on the NLRP3 inflammasome stimulation in macrophages shows contradictory results. Li et al. demonstrated that BRB berberine-mediated AMPK signaling resulted in the enhancement in inflammasome activation, pyroptosis, and IL-1β release in ATP-stimulated macrophages. These effects of BRB on macrophages were correlated with the increased bactericidal ability of macrophages both in in vitro and in vivo models [54,57]. However, another study showed the inhibitory role of BRB in triggering the NLRP3 inflammasome in human macrophages stimulated with ATP, ox-LDL, and serum amyloid A, thus inhibiting the secretion of IL-1β. Mechanistically, BRB was proposed to repress phospho-IκB expression and inhibit IκB degradation with the resulting effect on NF-κB pathway inhibition, attenuating the expression of the IL-1β precursor protein [58]. Similarly, in another study, BRB significantly reduced the expression of the NLRP3 inflammasome at the transcription and translation level, and decreased IL-1β secretion via partially inhibiting the activation the TLR4/Myd88/NF-κB signaling pathway in THP-1 macrophages stimulated with phorbol myristate acetate [59]. The repressive effect of BRB on NLRP3 inflammasomes in activated macrophages signifies its potential anti-atherogenic role in cardiovascular disease. Further, the effect of BRB in ameliorating insulin resistance by targeting the NLRP3 inflammasome was studied in LPS-primed macrophages stimulated with the palmitate–BSA complex to mimic in vitro adipose tissue macrophages (ATMs) with the activated NLRP3 inflammasome, producing high levels of proinflammatory cytokines. The study showed that BRB induced upregulated autophagy in ATMs via the AMPK-dependent pathway. This correlated well with the increased expression level of Beclin 1 and LC3B-II upon BRB treatment, with little to no effect on p62 expression. BRB also inhibited obesity-induced ATMs infiltration and NLRP3 inflammasome activation in adipose tissue, subsequently suppressing insulin resistance in a mice model fed with a high-fat diet [60].

#### 3.3.4. Neurological Disorders

The neuroprotective and anti-neuroinflammatory role of BRB evaluated in a Parkinson’s disease mice model treated with 1-methyl-4-phenyl-1,2,3,6-tetrahydropyridine (MPTP) showed that the BRB treatment induces enhanced autography, which promotes the autophagic degradation of NLRP3 and inhibits NLRP3 inflammasome activation, thus mitigating neurotoxicity [61].

#### 3.3.5. Nephropathy

Further, BRB also exhibits substantial anti-hyperuricemia and nephroprotective potential and has been shown to improve kidney function in diabetic and atherosclerotic conditions. The investigation of the nephroprotective role of BRB in a hyperuricemia mice model by Li et al. demonstrated that high BRB doses suppress the expression levels of NLRP3, ASC, caspase1, and IL-1β, thus inhibiting NLRP3 inflammasome activation, which correlated with the significant reduction in pro-inflammatory cytokine (interleukin [IL]-1β and IL-18) levels in serum and the kidney [62].

#### 3.3.6. Arthritis

The inhibitory effect of BRB in downregulating NLRP3 and IL-1β expressions was reported in monosodium urate crystal (MSU)-induced monocytes as an inflammatory model for gouty arthritis [63]. In another study, BRB showed the promising beneficial effect for the treatment of gouty arthritis in an arthritic rat model by activating the Nrf2 antioxidant pathway and suppressing the secretion of pro-inflammatory mediators, which overall reduced paw edema, pain score, and articular elastase activity in rats. An in-depth study demonstrated that BRB treatment suppressed the expression of NLRP3, IL-1β, caspase 1, and TXNIP, which correspond to the decreased level of pro-inflammatory cytokines IL–1β and TNF-α. Further, it was shown that BRB treatment upregulated the expression to Nrf2 transcription factor, along with the downregulation of its inhibitory subunit Keap1. Upregulated Nrf2 further promoted the transcription of its associated cytoprotective antioxidant enzymes: CAT, SOD1, HO–1, GPx, and NQO1, thus decreasing the intracellular oxidative stress [64].

#### 3.3.7. Viral Infection

Other than human metabolic disease, BRB also exhibited its potential role in viral pneumonia treatment. Liu et al. demonstrated the anti-inflammatory effect of BRB, which induced mitophagy and inhibited mitochondrial ROS generation, thus suppressing NLRP3 inflammasome stimulation and IL-1β release in virus-infected J774A.1 macrophages. BRB-induced mitophagy was evident from the increased and decreased expression of LC-3 II and p62, respectively, colocalized signals of LC-3 II with mitochondria and BNIP3, and autophagocytosed macrophages, partially mediated via the BNIP3-dependent pathway. The underlying anti-NLRP3 inflammasome mechanism resulted in the reduction in ROS generation along with the suppression of monocyte chemoattractant protein-1 (MCP-1) and TNF-α levels, thus inhibiting lung inflammatory injury in a pneumonia mice model infected with the H1N1 influenza virus [65].

Most of the anti-inflammatory mechanisms exerted by BBR treatment showed the involvement of several different signaling pathways. Zeng et al., for the first time, elucidated the direct binding target of BRB. Using a chemical proteomic-based strategy activity-based protein profiling (ABPP), BRB was shown to directly target the NEK7 protein. The molecular mechanism suggested that the binding of BRB with NEK7 blocks the NEK7−NLRP3 interaction, thus inhibiting further activation of the NLRP3 inflammasome, resulting in its anti-inflammatory efficacy [66].

Other than natural phytochemicals such as BRB, a class of endogenous inhibitors, known as specialized pro-resolving mediators (SPMs), are one of the most important naturally occurring lipid-derived mediators acting as pro-resolving anti-inflammatory molecules. These SPMs are involved in downregulating unwarranted inflammation caused by noninfection and tissue injuries, thus inhibiting the development of chronic inflammatory diseases [67]. SPMS families including lipoxins, resolvins, protectins, and maresins are enzymatically derived from essential omega-3 polyunsaturated fatty acids via cyclooxygenase and lipoxygenase pathways. SPMs are regarded as immunoresolvents because of their potent role as a dual anti-inflammatory and pro-resolving agent by restoring tissue vasculature, regeneration, and/or repair of injured tissues, along with resolving uncontrolled inflammation [68]. Furthermore, these SPMs have been shown to modulate the NLRP3 inflammasome pathway and reduce IL-1β expression and secretion in several inflammatory models such as in vitro LPS-induced primary human blood monocytes, THP-1 monocytic cells [69], oxidative stress-induced THP-1 macrophages [70], and in animal models of burn wounds [71], post-myocardial infarction [72], high-fat obesity [73], and NASH [74]. Studies have reported mechanistically various SPMs resolvin D2, resolvin D1 (RvD1), and 17S-hydroxy DHA acting via reducing ASC oligomerization, inflammasome assembly, and caspase-1 activity to inhibit both the priming and activation of the NLRP3 pathway [75,76]. Lee et al. further showed that the genetic deficiency of NLRP3 increases SPM lipoxin B4 in septic mice, and in LPS and ATP-stimulated macrophages, thus prompting the resolution of inflammation [77].

## 4. Conclusions

The uncontrolled and unregulated activation of immune responses leads to a pro-inflammatory cytokines storm and significantly increases the risk of several chronic human diseases. The overstimulation of the immune system is linked to dysregulated NLRP3 inflammasome activation. Few phytochemicals have been shown to modulate the NLRP3 inflammasome signaling with the subsequent suppression in levels of inflammatory mediators. BRB is one of the traditional Indian and Chinese phytomedicines extensively used for treating a broad spectrum of human diseases. However, recently, BRB has gained renewed interest because of its wide range of beneficial pharmacological effects and underlying mechanism(s). BRB has already been reported to exhibit anti-inflammatory effects via inhibiting NF-κB signaling; however, its role in controlling the NLRP3 inflammasome pathway is still not elucidated completely. Here, we have reviewed and discussed several studies demonstrating the potential of BRB in NLRP3 inflammasome suppression, along with its underlying but partially elucidated molecular mechanism(s), thus warranting in-depth exploration. This requires investigation and validation in more appropriate experimental disease models, which could be useful for the development of BRB as a novel and clinically applicable therapeutic agent against chronic inflammatory diseases.

## Figures and Tables

**Table 1:** Chronic inflammatory diseases and their associated NLRP3 inflammasome activators.

Disease	Activators/Stimulator
Obesity	Saturated free fatty acids, cholesterol crystals, ceramides, adipokines, and hyperglycemia
Type 2 Diabetes	Saturated free fatty acids, ceramides, high levels of glucose, uric acid, and Islet amyloid polypeptide
Atherosclerosis	Cholesterol crystals, calcium phosphate crystals, and oxidized LDL
Cardiovascular diseases ypertension, ischemic injury, cardiomyopathy, and myocardial infarction)	Low-density lipoprotein (LDL), cholesterol crystal, external irritants, and HIV-1 infection
Liver Disease (Alcoholic and Nonalcoholic steatohepatitis and Viral hepatitis)	Cholesterol crystals, ethanol, nanoparticles (rare-earth oxide (REO), quantum dots, and mesoporous silica), and hepatitis C virus (HCV) infection
Parkinson’s Disease	Aggregated α-synuclein
Alzheimer’s Disease	Amyloid-β plaques, Tau monomers, and oligomers

**Table 2:** Potential mechanisms of several classes of NLRP3 inflammasome inhibitors with examples [33,34].

Inhibitor Class	Examples	Potential Mechanism(s) of Inhibition
**Direct Inhibitors**		
**Small** **Molecules**	MCC950	Binds and inhibits the ATPase activity NACHT domain, thus hindering NLRP3 oligomerization.
	3,4-Methylenedioxy-β-nitrostyrene (MNS)	Binds to the NACHT and LRR domains, thus inhibiting ATPase activity and NLRP3 oligomerization.
	CY-09	Binds and inhibits ATPase activity of NACHT domain and hinders NLRP3 oligomerization.
	(N-[3′,4′-dimethoxycinnamoyl]-anthranilic acid (Tranilast)	Binds to NACHT domain and blocks NLRP3 oligomerization.
	OLT1177	Impedes ATPase activity and obstructs NLRP3 oligomerization.
	Oridonin	Binds cysteine 279 of NLRP3 NACHT domain via a covalent bond, blocks NLRP3-NEK7 interaction, and hinders subsequent NLRP3 inflammasome activation.
**Indirect Inhibitors**		
**Small** **Molecules**	Glyburide	Inhibits ATP-sensitive K^+^ channels and blocks ASC aggregations.
	16673-34-0	Interferes with the NLRP3 protein activation and/or the aggregate with the ASC scaffold, and blocks inflammasome activation.
	JC124	Downregulates NLRP3, ASC, caspase-1, pro-IL-1β, TNF-α expression; and inhibits inducible nitric oxide synthase (iNOS).
	FC11A-2	Hinders autocleavage of procaspase-1 and diminishes activated caspase-1 level, and suppresses IL-1β/18 release.
	Parthenolide	Induces cysteine modifications and inhibits caspase-1 activation and ATPase activity of NLRP3 protein.
	VX-740 and VX-765	Blocks caspase-1 activation by covalent modification of the active site catalytic cysteine and resultant cleavage of pro-IL-1β/18.
	Bay 11-7082	Modifies cysteine residues of ATPase site of NLRP3, and inhibits APTase activity of NLRP3 and ASC organization.
	β-Hydroxybutyrate (BHB)	Inhibits K^+^ efflux, inhibits ASC oligomerization, and suppresses IL-1ß and IL-18 production.
**Interferons**	IFN-α and IFN-β	Phosphorylates STAT1 transcription factor, suppresses NLRP3 inflammasomes, and inhibits caspase-1-dependent IL-1β activation. Induces STAT-dependent IL-10 production and reduces pro-IL-1α and pro-IL-1β levels.
**Autophagy Inducers**	Resveratrol	Induces autophagy, suppresses mitochondrial damage, and inhibits NLRP3 inflammasome activation.
	Arglabin	Induces autophagy, reduces inflammation and cholesterol levels, and inhibits NLRP3 inflammasome activation.
	Cannabinoid receptor 2 (CB2R)	Induces autophagy and inhibits NLRP3 inflammasome priming and activation.
**MicroRNAs**	MicroRNA-223	Binds to the 3′ UTR of the NLRP3 transcript, suppresses NLRP3 protein expression, and prevents priming of NLRP3 inflammasome and IL-1β secretion.

**Table 3:** Major plant family and representative plant species and their parts as sources of berberine.

Family	Plant Species	Used Parts
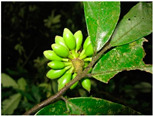 ***Annonaceae***	*nnickia polycarpa*	Bark
	*nickia chlorantha*	Bark
	*Annickiapilosa*	Bark
	*Rollinia mucosa*	Fruits
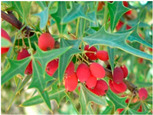 ***Berberidaceae***	*Berberis vulgaris*	Stem, root
	*Berberis aristate*	Bark, root, stem, fruits, extract
	*Berberis petiolaris*	Root
	*Thunbergia*	Stem
	*Aquifolium*	Root
	*Asiatica*	Bark, root, stem
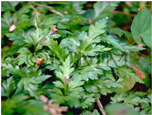 ***Ranunculaceae***	*Coptis Chinensis*	Root
	*Coptis teeta*	Rhizome
	*Coptis japonica*	Rhizome
	*Hydrastis canadensis*	Whole plant
	*Xanthorhiza simplicissima*	Roots, stem, leaves
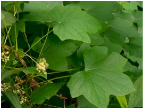 ***Menispermaceae***	*Tinospora sinensis*	Stem
	*Tinospora cordifolia*	Stem
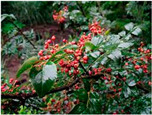 ***Rutaceae***	*Zanthoxylum schreberi*	Stem, branches
	*Zanthoxylum armatum*	Stem
	*Phellodendron lavallei*	Bark
	*Phellodendron amurense*	Barks, branches, leaves
	*Evodia meliaefolia*	Bark
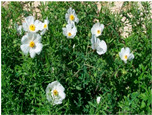 ***Papaveraceae***	*Argemone albiflora*	Aerial parts, roots
	*Argemone mexicana*	Epigeal parts, leaves, seeds, roots, fruit capsules, latex
	*Argemone ochroleuca*	Seeds
	*Argemone squarrosa*	Aerial part

## Data Availability

Not applicable.

## Sample Availability

Samples of the compounds are not available from the authors.
